# Embedding the living well toolkit into service delivery – A complex process

**DOI:** 10.1016/j.pecinn.2022.100033

**Published:** 2022-03-26

**Authors:** Suzie Mudge, Nicola Kayes, Deborah Payne, Greta Smith

**Affiliations:** Centre for Person Centred Research, Auckland University of Technology, Private Bag 92006, Auckland 1142, New Zealand

**Keywords:** Implementation, Person-centred, Toolkit, Communication

## Abstract

**Objective:**

To embed the Living Well Toolkit package and to understand how it was implemented at each site and to explore the experiences of users.

**Methods:**

The toolkit package was introduced in four rehabilitation settings using a tailored implementation process negotiated with each site. The varied data sources were analysed drawing on directed content analysis.

**Results:**

Clients with neurological conditions and clinicians initially weighed the merits of the toolkit package. A positive weighing up was prerequisite for deciding to use. Clinicians described considerable *thought and planning* to make the toolkit package *fit and flow* in clinical practice. Users of the toolkit package described ways in which it shaped their thinking.

**Conclusion:**

Implementation of the toolkit package was a complex process for clinicians and services, involving ongoing work to optimise its impact relative to the client and context. Clinicians and clients who used the toolkit package described positive changes, congruent with person-centred communication.

**Innovation:**

The Living Well Toolkit is freely available for all to use. Clinicians who used reflective and responsive thinking to make the toolkit package work found it provided them with a broader perspective of the client.

## Highlights

• Tailoring interventions to meet service needs takes time and effort.
• Clinicians work hard to successfully implement person-centred tools into routine practice.
• Reflexive practice is a key feature clinicians use to fit a new tool into practice.
• The Living Well Toolkit can be used by services to enhance person-centred communication.

## 1 Introduction

A change in how we work with people living with neurological condition and their families is needed to address findings indicating people’s long-term recovery and adaptation is often made difficult by a system that struggles to deliver quality care [[1], [2], [3]]. Person-centred communication, the utilisation of people’s expectations of care [4] and their strengths and capacities [5] are frequently not translated into routine practice [2,9], and services are often un-coordinated [[6], [7], [8]]. Importantly, studies have suggested that relatively simple changes in how we listen to and work with people living with a long-term neurological condition can improve their outcomes, quality of care and assist them to take charge of their condition resulting in improved health [[9], [10], [11], [12]].

**Fig. 1:**
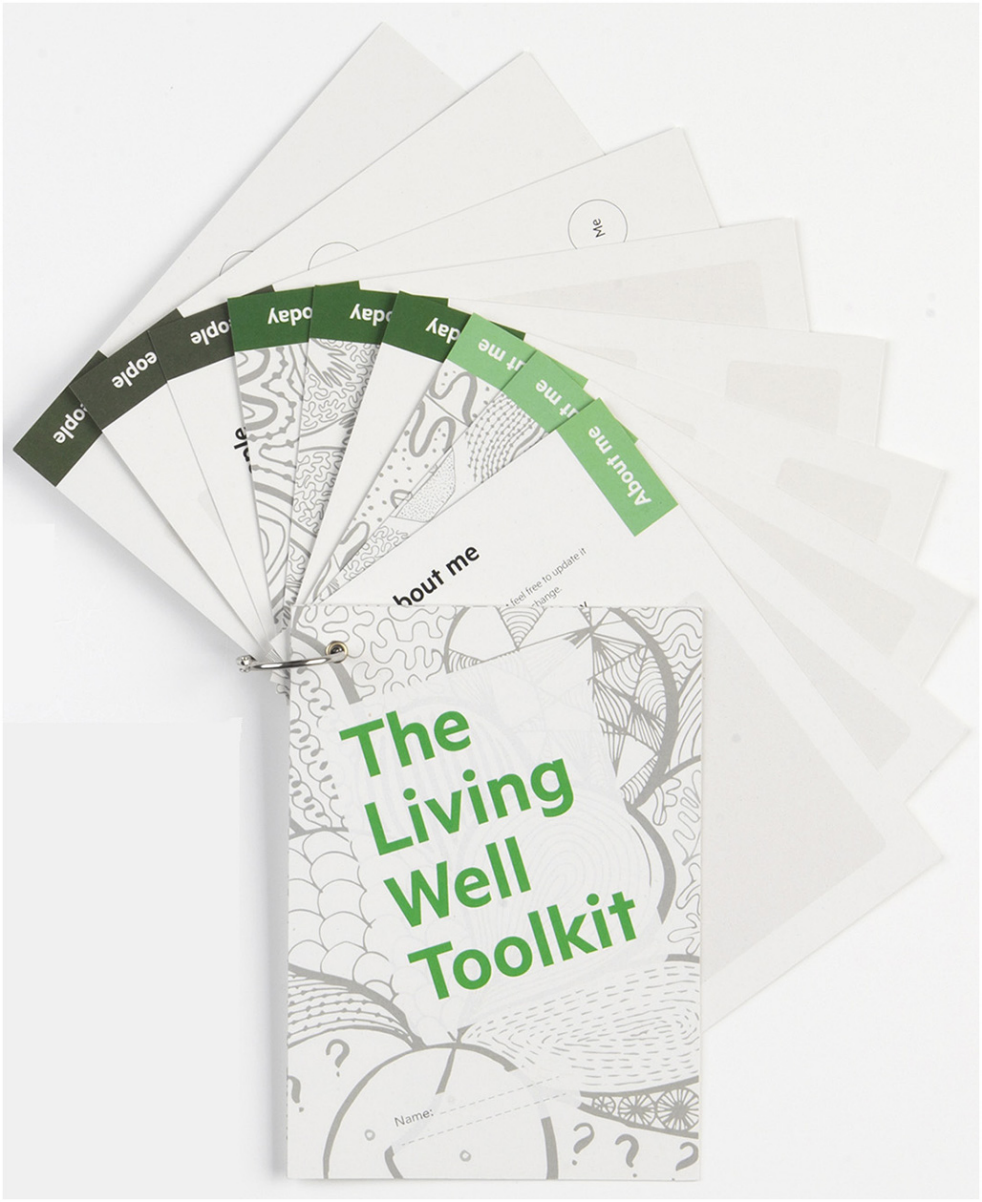
Client toolkit.

**Fig. 2:**
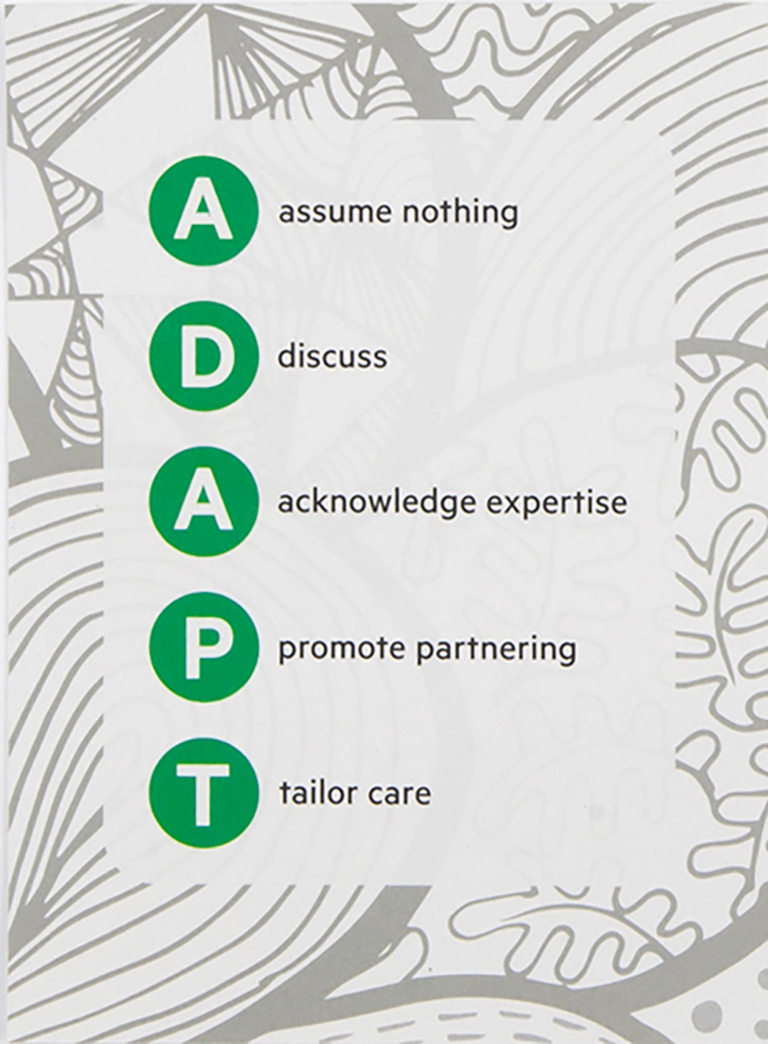
ADAPT portion of clinician’s resource.

The overarching aim of the project was to operationalise three processes (person-centred communication, harnessing of people’s strengths and resources, and coordination of healthcare services across the lifespan) by developing a toolkit. In prior phases we developed and piloted the Living Well Toolkit package [13,14] (henceforth called the toolkit package), comprising two components. The first is a paper-based toolkit (Fig. 1) with three sections ‘About me’, ‘My needs today’ and ‘People’, designed to be held by the client and used in healthcare interactions as desired (henceforth called ‘client toolkit’). The second is a clinician’s resource that provides a structural support for clinicians to promote a way of working consistent with the client toolkit and the three core processes. Themes from earlier phases of this work underpinned five key principles constructed into an acronym - ADAPT (**A**ssume nothing, **D**iscuss, **A**cknowledge client expertise, **P**romote partnering, **T**ailor care) to guide practice (Fig. 2). This was produced as a prompt card with a central orienting question ‘Who is this person and what do they need from me today?’ [13].

**Fig. 3:**
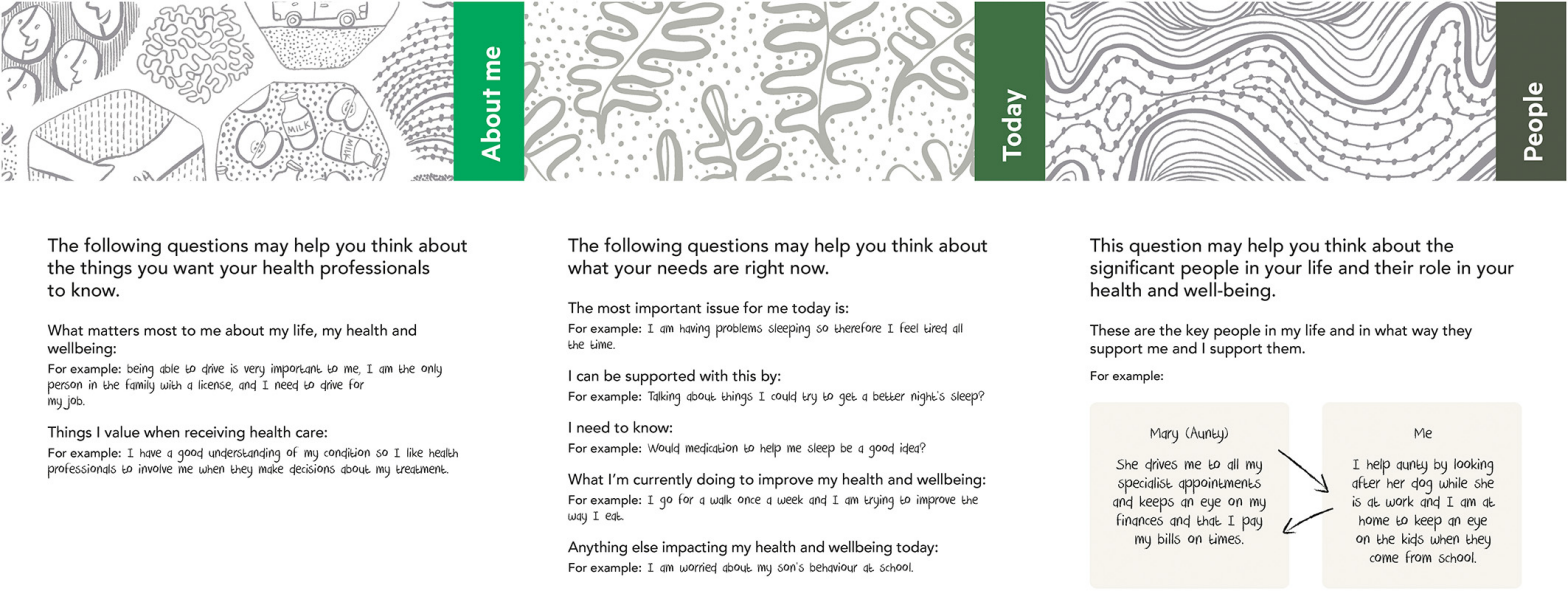
Client toolkit: prompt version.

We previously piloted the toolkit package in four rehabilitation settings to explore its utility and acceptability and related implementation processes [14]. In brief, although the philosophy of the toolkit package resonated with the values of clients with neurological conditions and clinicians, barriers limited the time, energy and work clinicians were able or prepared to invest, so that uptake of the toolkit package was modest. Analysis informed revisions to the toolkit package, including simplification of the client toolkit (Fig. 3) and development of a training package for clinicians [14]. The training package included three modules (bronze, silver and gold), which were intended to be interactive and reliant on discussion with and reflection from participants. Each module was a pre-requisite for subsequent modules. A series of training videos were developed to be used in conjunction with the modules. The refinements and training package were intended to improve sense-making and minimise the cognitive barriers [14] to optimise implementation in a final phase (the focus of this paper).

### 1.1 Aim

The aim of this implementation phase was to embed the refined toolkit package in routine service delivery. We wished to understand how the toolkit package was implemented in each setting and to explore users’ experiences of the toolkit package.

## 2 Methods

### 2.1 Design

We used an acceptability-implementation hybrid design drawing on qualitative methods [15]. Consistent with a knowledge translation approach [16], we focused on adapting the intervention to each context, identifying and promoting facilitators and assessing and responding to barriers.

### 2.2 Setting

We aimed to recruit six health and social services working with people with long-term neurological conditions to implement the toolkit package into their routine practices.

### 2.3 Intervention

**Fig. 4:**
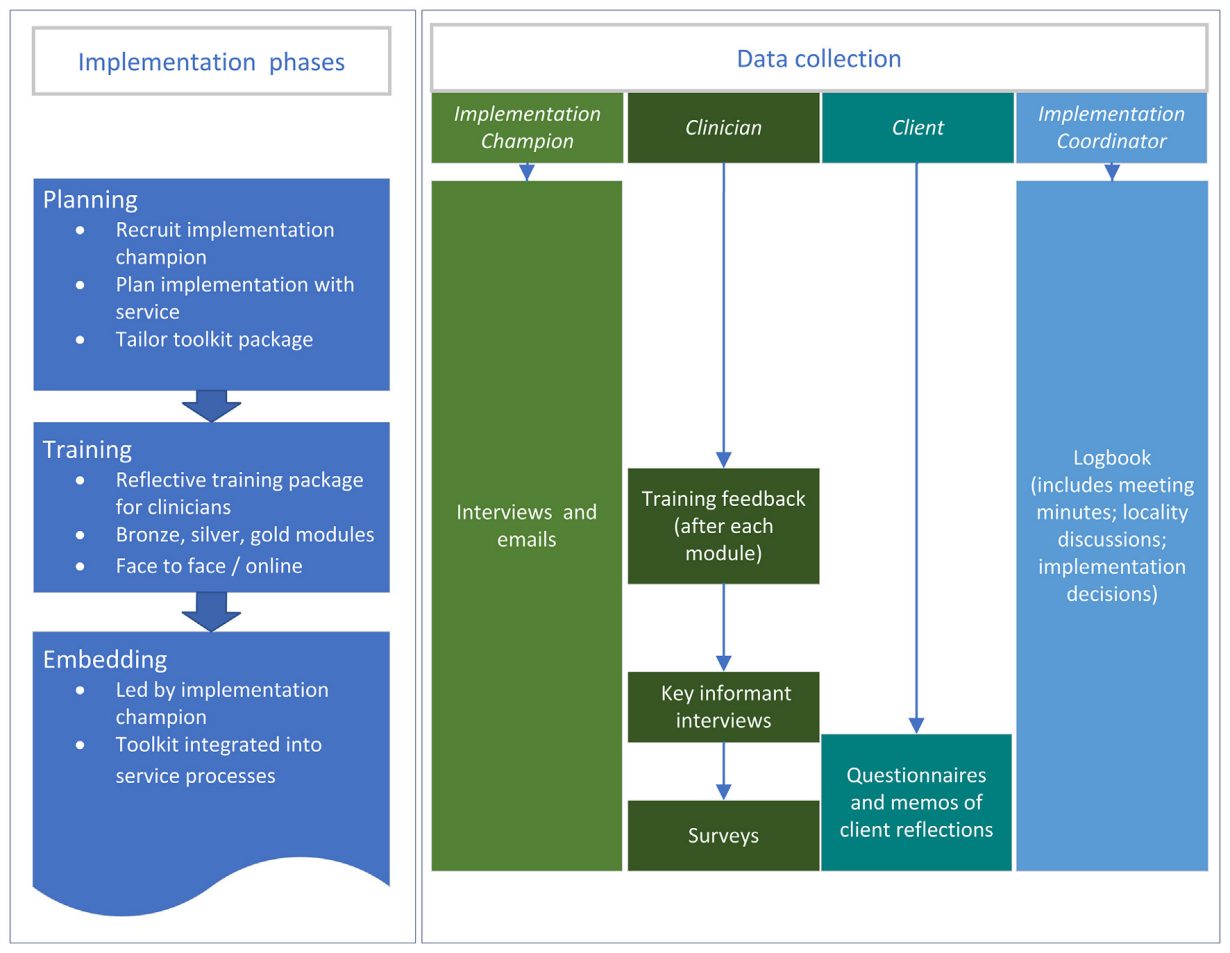
Implementation phases and related data collection.

We worked with each service to develop an implementation plan in collaboration with an implementation champion appointed from within the service (Fig. 4), whose role was to promote the use of the toolkit amongst staff. Together, we customised the toolkit package to enable integration into existing service processes while retaining the core principles and intended purpose of the toolkit. We offered a reflective training package for staff at each facility [14].

In our interactions with implementation champions and clinical staff, we emphasised the flexible nature of the toolkit package to suit the needs and preferences of clients. We anticipated that clinical staff would routinely use principles from the clinician’s resource to underpin their interactions and additionally would introduce the client toolkit to most of their clients. The timing of introduction of the client toolkit was left to the discretion of clinical staff.

### 2.4 Participants

We aimed to recruit clients, clinicians or support workers involved in their care and implementation champions from each setting to share their experiences of interaction with the toolkit package.

#### 2.4.1 Implementation champions

Each service appointed one implementation champion from the clinicians already working in the setting to lead the implementation of the toolkit package and work with the research team to customise the implementation process to meet service-specific needs. They were invited to share their experiences and reflections of the implementation process.

#### 2.4.2 Clinicians and support workers (hereafter referred to as ‘clinicians’)

We invited all clinicians from each setting to provide feedback irrespective of their direct use of the toolkit package and/or completion of training modules. This was because, at two of the sites, all clinicians had at least some exposure to the toolkit package as the implementation champions had embedded it into routine processes. We asked the implementation champion in each setting to identify two clinicians from their setting who were willing to additionally participate in an individual key informant interview of up to 60 minutes. The implementation champions specifically sought a clinician who had reported a positive interaction with a client related to use of the toolkit package and a clinician who had experienced challenges when using the toolkit.

#### 2.4.3 Clients

We aimed to recruit up to 25 consecutive consenting clients from each facility. Clients were included if they had a long-term neurological condition, were over the age of 18 and had received healthcare services at one of the participating facilities following implementation of the toolkit package. Clients were invited irrespective of their exposure or use of the client toolkit as we were interested in exploring their experience of care given the potential of the toolkit package to contribute to changes in service delivery without its use being made explicit to clients. Clients were Invited through one of two mechanisms: an invitation tab attached to the front page of the client toolkit invited clients to contact the research team if willing to participate. For those clients who did not receive a client toolkit, clinicians invited their participation and if willing, passed their contact details to the research team.

Clinicians who participated in informant interviews outlined above identified clients who were also then invited to share their perspective of the clinical interaction supported by the toolkit package.

### 2.5 Procedures

On completion of the training modules, services took at least three months to embed the toolkit package into routine service delivery. Our implementation coordinator (member of research team) led this process and provided support to the service in coordination with the local implementation champion.

### 2.6 Data collection

We collected data from four different sources (implementation champions, clinicians, clients, implementation coordinator) in a range of formats (Fig. 4). All data were in the written format, with the exception of interviews, which were audio recorded and then transcribed using intelligent verbatim.

#### 2.6.1 Implementation champions

The study implementation coordinator interviewed each service implementation champion at least twice for a maximum of 20 minutes per occasion. The purpose was to collect information on the activities and strategies the implementation champions adopted to support implementation, key decisions, challenges and tensions and their general reflections on the process. This data was supplemented by written documentation exchanged between parties relevant to the implementation process.

#### 2.6.2 Clinicians

We invited clinicians to provide feedback about the content, relevance and usefulness of the training modules through feedback forms. At least three months into the embedding phase, the implementation champion distributed an online survey to all clinicians in their facility to capture acceptability and perceived impact of the toolkit package.

Interviews with nominated clinicians explored in-depth identified examples of toolkit use, including key features of those exemplar situations and perceived impact on implementation.

#### 2.6.3 Clients

All clients were invited to complete a questionnaire about their experiences of interactions with their key clinician, along with an open-ended survey about their exposure to and use of the client toolkit. We used memos to capture reflections offered by clients during survey administration.

#### 2.6.4 Implementation coordinator

The implementation coordinator maintained a logbook to detail activities related to implementation of the toolkit package at each site. Activities included training, meetings between researchers, implementation champion and clinical staff at localities. Meetings were scheduled prior to and over the implementation period as needed and were specific to culture, processes and issues at each site. The implementation coordinator was asked to record any issues that arose, strategies employed and local decisions made.

### 2.7 Analysis

We coded data from the multiple sources line by line using directed content analysis [17] to answer the two research questions: a) how was the toolkit package implemented? and b) what are the perceptions of those exposed to the toolkit package? After data familiarisation, we developed the initial coding framework drawing on broad data categories, shown in the appendix. We started with 31 codes, each relating to one or more of the categories. We revised and refined the codes as analysis proceeded by adding and collapsing codes. We grouped related codes to form themes that related to the two research questions.

## 3 Results

### 3.1 Settings

Four settings implemented the toolkit package into routine practice. Basic characteristics of each setting are shown in Table 1. The three clinical services each appointed an implementation champion but one, a support agency, elected not to as the support workers had a culture of working autonomously and had little contact with each other. Thus, there were three sets of interviews from the implementation champions together with documentation related to implementation decisions at each locality. Sixteen clinicians provided feedback on the training. Twelve clinicians (eight physiotherapists, two occupational therapists, one speech language therapist, one nurse) completed the online survey and three clinicians participated in interviews. Fifteen clients responded to the open-ended survey and eight memos recorded the discussion between client participants and the researcher. None of clients approached for an interview agreed to participate.

**Table 1:** Characteristics of implementation settings.

Settings				
Context	Community rehabilitation clinic (neurorehabilitation)	Residential rehabilitation clinic (neurorehabilitation)	Community rehabilitation clinic (neurorehabilitation)	Support Agency for a specific neurological condition
	City	City	Small city servicing rural surrounds	City
	Multidisciplinary team with 10 staff members	Multidisciplinary team with 37 staff members	Multidisciplinary team with 31 staff members	Charitable organisation providing support via support worker visits. 1 general manager and 3 support workers.
Training*	3 modules delivered in 3 sessions	3 modules delivered in 3 sessions	3 modules delivered in 3 sessions	3 modules delivered in 2 sessions
	Attendance numbers: Bronze – 8 Silver – 5 Gold - 3	Attendance numbers: Bronze – 11 (module formed part of staff induction) Silver – 19 (different staff members to bronze module) Gold – 12 (same staff members as silver module)	Attendance numbers: Bronze – 31 (all teams) Silver – 18 (rehabilitation team only) Gold – 10 (same as silver module)	All four staff attended both sessions.
Implementation Process	Philosophy of toolkit package was explicitly introduced in new client welcome pack	Client toolkit separated into three parts and integrated into three different established processes. Some changes made to wording.	Some of the content of toolkit extracted and inserted into assessment forms, which were compulsory for staff to use.	Toolkit viewed as a resource to be issued if appropriate
	Individual clinicians responsible for introduction of client toolkit	Although clients may have interacted with one or more of the parts, they did not see client toolkit in entirety	Client toolkit not physically introduced to any clients.	Individual support workers responsible for introduction of client toolkit.

Notes: * For more details about the training modules, please refer to Mudge et al, 2020 [14].

### 3.2 Themes

**Fig. 5:**
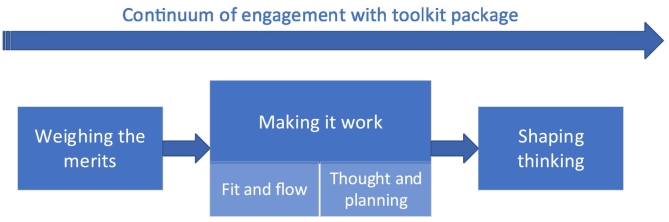
Themes related to the continuum of engagement with the toolkit package.

We constructed three themes which represent a general sequence of interaction with the toolkit package (Fig. 5). The first theme **Weighing the Merits** describes the initial assessment made by clients and clinicians of the toolkit package. A positive weighing up appeared to be a prerequisite for **Making it Work** (the second theme). Clinicians described considerable *thought and planning* to make the toolkit package *fit and flow* in service delivery. The final theme **Shaping Thinking** describes the changes depicted by clients and clinicians through their use of the toolkit package. Each of these themes and subthemes represent decision junctures at which individuals continue or cease interacting with the toolkit. Examples of decisions to engage or not are discussed in more detail below.

#### 3.2.1 Weighing the merits

The starting point for clinical services was recognition that the toolkit package aligned with their philosophy of practice. This decision was usually conceptual rather than practical. Services also valued the toolkit package as a structure to support service initiatives:

‘*I guess that whole idea about the client being at the centre and client making the choices and the client being the expert and being self-managing and being heard by the therapist and the professionals working around the client as opposed to the client having to fit in with the systems or the structure of how it was done in the unit. It just fitted with what we were achieving or trying to achieve at [our service] just working a whole lot more flexibly and not just paying lip service to the idea of being client centred*.’ (Service manager)

Organisational support was critical for buy-in to implement the toolkit package, however, was not sufficient alone to achieve buy-in. Clinicians individually assessed the toolkit package, many recognising its alignment to person-centred care. For some clinicians this meant they did not perceive it to be different from their current practice:

*It’s not information that I think we are not covering anyway and I think that’s probably why it’s not getting used is because we feel like we are addressing it already.* (Service provider)

In this example, the toolkit package was not perceived to add value to the current way of working. There were other instances where it was perceived to duplicate information gathered through other tools already in use. Some clinicians viewed the toolkit package as another resource*,* competing with other methods of supporting person-centred practice. Such responses appeared to lead to dismissal of and disengagement with the toolkit package.

Similarly, some clients perceived there was no need for the client toolkit, particularly if they worked with clinicians who they perceived knew what mattered to them and with whom they had good communication. A few clients dismissed the toolkit because they did not see the toolkit to be consistent with their personality or style of communication:

*…putting pen to paper was not something I was going to do.* (Client)

Although some clinicians and clients did not engage with the toolkit package past this initial assessment point, others who found it aligned with their thinking, were prepared to invest time and effort into **making it work**. This second theme, **making it work** also describes services who engaged in an ongoing process of implementation to promote the toolkit package, encouraging and involving staff in a way that accounted for the service context and allowed staff to progress at their own pace in adopting new habits. **Fit and flow** was an important aspect of **making it work** and describes the logistics and mechanics of making the toolkit package work. Firstly, at a service level this entailed fitting the toolkit package with established processes, timing its introduction relative to the clinical pathway, identifying which staff member was most appropriate for introduction, as well as more practical considerations, such as how to *chop and change* (implementation champion) the adaptable aspects of the toolkit package to optimise uptake by clinical staff. The negotiation between each service and the research team was tailored to the service’s needs and attempted to be responsive to perceived barriers. Small changes to the toolkit package and the implementation process continued throughout the course of the implementation period in consultation with each service. Clinicians also considered **fit and flow** at an individual client level. Even though clinicians became more familiar with the toolkit package and learnt from the implementation process, they described still having to work to make it **fit and flow** for each client. This involved considering the logistics and mechanics, such as session structure and context and the timing of introduction. In the following quote, a clinician contrasts two different occasions of introducing the toolkit.

*Because there were discreet circumstances when it was real to introduce it with these forthcoming significant appointments for these people; that seemed very appropriate to use at the time to do that. Where it didn’t seem to work for me was when I tried to introduce it cold, “Just have a think about your interactions with your health professionals.” And I got a bit blanked really because it was like, “Where does this come from?” and I think it did feel like I was trying to overlay something in addition to what we were trying to achieve on the day for that client.*

The clinician became aware that how and when she introduced the toolkit made a difference to the client’s perception of utility of the toolkit. Clinicians felt some pressure to get the introduction right so the toolkit did not feel like an add-on to the session.

The other main component of **making it work** was **thought and planning**, which represents the mental work clinicians described to prepare for use of the toolkit package clinically. Thought was also required in response to a client’s reactions and behaviours. The data shows that clinicians considered each client and their context and used proactive, reactive and reflective aspects of clinical reasoning. Clinicians used their professional judgement to identify optimal situations for use of the toolkit package, particularly relative to the introduction of the client toolkit. They grappled with questions such as, Who is the ideal client? When is the best time to introduce it? How do I describe it?

*Yeah… so it’s kind of knowing when to introduce that [the client toolkit], because we do the same if we are doing fatigue management and then bring out some resources on that. It’s bringing them to [the session] and, ‘This is what we are focusing on that this session.’ at this time, when they can take it in. So, it’s weighing up not overloading them and, ‘Can they take it?’ versus, ‘It’s going to be too much for them’. (Clinician)*

In coming to answers to these questions, clinicians considered aspects such as the client’s cognitive capacity, personality, ability and context. Clinicians also showed flexibility by using the questions of the client toolkit within their sessions to test whether they had accurately judged the timing and response of the client. While this level of clinical reasoning was predominant in the clinician data, proactive **thought and planning** was also evident at a service level, for example in deciding when and how to introduce the client toolkit within the continuum of care.

Clients emphasised trust as a critical factor for them to share information with clinicians. From their perspective, trust was the important element in **making it work**. As one client phrased it, ‘*All depends on trust.’* Trust was also needed in those around them to keep what was written in the client toolkit private. The paper-based medium of the client toolkit was raised several times in relation to trust as it was perceived to be more difficult to secure than an electronic version.

Clients and clinicians both described aspects of **Shaping Thinking** through their use of the toolkit package. ‘*Makes you think’* is a quote from a client describing the impact the questions in the client toolkit had on him and typifies what use of the toolkit package made possible for him. Clients elaborated that the questions in the client toolkit also helped clarify their thoughts. In some cases, this triggered a focus on the future for some clients, particularly of broader needs than health alone: ‘*keeps health in focus but opens up more than that*’ (client). Clinicians and clients perceived that the client toolkit fostered control for clients that used it. One client explained, ‘*It [the client toolkit] helps you get something positive out of it [session with a clinician].* Clinicians described a broadening of their perspective and a better understanding of what was important to the client and expressed those clients who used the client toolkit were more easily able to communicate their needs through the structure provided.

*I have clients who now feel empowered to seek their own answers from their GPs and specialists. They also question aspects of the work that I do with them and I encourage them to do this*. (Clinician)

One clinician described a client who used the client toolkit to prepare for a needs assessment and consequently had a more positive experience than he had previously:

*I was delighted actually and I think it was really important for this person because my impression is that they weren’t usually treated as an expert and weren’t usually listened to and went with the flow with however the health professional decided things were going to go. (Clinician)*

In some services, the client toolkit was not visible to clients, where clinicians had embedded the questions from the client toolkit into their practice instead of using the client toolkit itself. They perceived the questions were beneficial and augmented their practice. They noted that the questions were broader than those they typically asked, which led to a better understanding of the client and their context. Clinicians particularly valued the insight they gained into what mattered to their client. For example, a clinician contrasted her experience when using the toolkit questions and what that enabled, compared to her previous way of working:

*… get a better idea of what the client really wanted out of their therapy as a whole; what the end picture looked like to him, more than the impairment/activity focused discussions we would often have [previously]. (Clinician)*

Clinicians felt a more collaborative partnership was facilitated by the toolkit package, including forging a stronger therapeutic relationship. One implementation champion promoted the use of the toolkit package for clinicians who expressed difficulty building rapport with clients. Clinicians also identified the toolkit package had reduced their use of assumptions and helped them build partnerships with clients. Consequently, clinicians felt they had a better understanding of the client's values, perspectives and needs.

Many clinicians recognised the toolkit package resonated with their philosophy of practice and as such, acted as a reminder of the importance of person-centred practice. Clinicians gave examples of how the toolkit package provoked a heightened consciousness in clinical interactions:

*I think sometimes the concepts still come up when I am doing my daily therapy anyway and I notice I am more conscious with checking in with a patient or a client about what’s the most important issue for them as opposed to just assuming that it was just like what we’d said a few months back.* (Clinician)

There was evidence that, for clinicians, the toolkit package provoked ongoing reflective practice:

*The training and the resources are extremely useful; they keep me alert to my practice and encourage me to reflect on continuing and managing a client centred approach with clients.* (Clinician)

## 4 Discussion and conclusion

### 4.1 Discussion

Our findings indicate the toolkit package needed to pass many decision junctures to be successfully used (Fig. 5). Initially clinicians and clients weighed the merits and proceeded only if they anticipated some benefit. When clinicians decided to engage further, they demonstrated thoughtful reflection and work to incorporate the toolkit package into routine practice [18,19]. Most of those who did use the toolkit package, used aspects of it rather than it in its entirety. Those who did not use the toolkit package did not necessarily dismiss it initially or at all. For some, it was not used because of other priorities or other ways of achieving a similar result; generally careful thinking was evident in this decision process. These are important findings as researchers often attribute low uptake and implementation challenges to clinicians and services [14,20,21], which may not do justice to the work that clinicians, in particular, do to consider whether and how to use a tool and then to optimise the potential of a tool for a particular client in their unique context. We recognise, however, that such a view does not account for pre-existing assumptions clinicians may hold in terms of for whom and under what circumstances a tool might be optimally integrated into practice (if at all). A challenge for researchers and clinicians implementing tools for person-centred practice is recognising that the reasons clinicians may not take up these tools is nuanced. It is important to recognise clinicians as thoughtful and reflexive and motivated to make tools work while also acknowledging that clinicians may hold inherent assumptions within their clinical reasoning processes [[21], [22], [23], [24], [25]] which have the potential to limit who can benefit. This implementation challenge is perhaps particular to person-centred practice where tools have the potential to embed fixed assumptions [26], which may be problematic given the uncertainties and tensions inherent in implementation [18].

Clinicians described ongoing work they needed to do to make the toolkit fit and flow, even when using the toolkit package repeatedly. Because each client used the toolkit as best suited their needs, clinicians’ learning and experience with previous clients may have had little relevance to subsequent clients, necessitating a high level of cognitive participation [27,28]. Despite refinements to the toolkit package to improve coherence [14], flexibility of use [29] remained a key feature consistent with person-centred practice [26] but likely contributed to the sense of ongoing work. We also recognise the range of drivers in service delivery [19], which may have competed with implementation of the toolkit and likely added to the work for clinicians to determine what should be prioritised for the particular context [30].

Data from individual clinicians and the implementation champions showed evidence of reflexive practice, previously identified as a critical step in making changes to clinical practice [12,23]. Some clinicians reported positive interactions and/or outcomes with clients which they attributed to use of the toolkit package. Positive experiences often act as validation of the legitimacy of the new approach to clinicians [[21], [22], [23]] and may even provide evidence to ‘sceptical’ colleagues of the merits of an intervention [31], however, we did not observe this effect. This difference may be due to modest uptake of the toolkit package, the relative isolation of clinicians in two of the services due to geographical spread and structures relating to contracting in the private sector.

Although the client toolkit was not widely used by clients in this implementation study, we designed the toolkit package in collaboration with clients to incorporate features that highlight client strengths and resources and promote planning for long-term care [13] We recognise that the client toolkit needed to be in the hands of clients for this to be achieved and we argue that clients are in the best position to decide if, when and how to use the toolkit [13]. We consider that making the toolkit freely available to clients [32] gives more control to clients who wish to use such a tool.

We believe that the negotiation with each service was critical to implementation. As a research team, we underestimated the amount of time and the extent of change required to make the toolkit package fit the needs and processes of each setting. We have deliberately avoided making assessments regarding how successful implementation was in each site for two reasons: a) we purposefully selected diverse implementation sites. As such, the unique and particular features of each site make comparison across sites difficult.; and b) each site tailored implementation to ensure fit within their service culture, needs and processes and so any pre-conceived ideas we had about what might constitute successful implementation were challenged through the course of this research to the extent that it did not feel appropriate to make judgements about whether one approach was more successful than another.

We acknowledge this study has limitations. While the pragmatic and participatory approach to implementation was a strength in terms of being able to attend to the unique needs of each participating service, it led to a variability in implementation approach and reach across services which made sampling, data collection and interpretation complex.

Although clinicians were positive about the ADAPT principles of the toolkit package, they identified limited opportunities or need for toolkit introduction to clients. Instead, the client toolkit questions were integrated into clinical templates in one service and were produced as separate worksheets in another. This meant that clients in these services did not have an opportunity to engage with the full client toolkit so did not interact with it as originally conceptualised. We believe the invisibility of the client toolkit to clients was further compounded by our research process of using clinical services as the mechanism to introduce the client toolkit. We acknowledge this was philosophically contradictory to the intent of the toolkit as it reinforced clinical control [12,19] precluding, for the most part, client ownership of the toolkit (and indeed, the decision to use it, or not).

The themes draw heavily on the experience of clinicians as limited distribution of the client toolkits to clients resulted in a small pool of potential client participants, none of whom agreed to participate in an interview. Client interviews would have potentially contributed to a richer understanding of the client perspective than the surveys did. We recognise this as a further unintended consequence of our research process.

### 4.2 Innovation

Although we conceptualised the clinician’s resource and client toolkit as a package, these components can be used separately and are freely available [32]. Clinicians readily integrated the clinician’s resource without reference to the client toolkit, highlighting the principles can stand alone and have the potential to shift clinicians towards a greater degree of person-centred communication. Clinicians who used reflective and responsive thinking to make the toolkit package work found it provided them with a broader perspective of the client.

## 5 Conclusion

Implementation of the toolkit package involved many decision points for all users. Alignment with values was needed to initially engage but the implementation of the toolkit package was a complex process for clinicians and services that involved ongoing work to optimise the impact of the toolkit package for the client and context. Clients emphasised trust as an important factor, which influenced their use. Clinicians and clients who used the toolkit package described positive changes, congruent with person-centred communication.

## Funding

This project was funded by the 10.13039/501100001505Health Research Council of New Zealand (grant number: 13/285).

## Ethics, consent and permissions

This study was approved by Auckland University of Technology Ethics Committee (AUTEC): 17/335. All participants provided written informed consent.

## CRediT authorship contribution statement

**Suzie Mudge:** Conceptualization, Methodology, Formal analysis, Investigation, Writing – original draft, Writing – review & editing, Funding acquisition. **Nicola Kayes:** Conceptualization, Methodology, Formal analysis, Investigation, Writing – review & editing, Funding acquisition. **Deborah Payne:** Formal analysis, Investigation, Writing – review & editing. **Greta Smith:** Formal analysis, Investigation, Writing – review & editing, Project administration.

## Declaration of Competing Interest

The authors declare that they have no known competing financial interests or personal relationships that could have appeared to influence the work reported in this paper.

## Figures and Tables

**Table:** 

Overarching categories
1. Initial implementation drivers at an organisational level 2. Implementation decisions and their impact 3. Factors impacting clinician engagement with the toolkit package 4. Factors impacting client engagement with the client toolkit 5. Implementation challenges 6. Perceived value and benefit to users of the toolkit package
